# Associations between blood ethylene oxide levels and serum neurofilament light chain concentrations in adults: evidence from the NHANES

**DOI:** 10.3389/fpubh.2025.1498919

**Published:** 2025-02-21

**Authors:** Shikun Li, Wenchen Li, Jis Huang Liu, Jing Ma, Yan Juan, Bo Chen

**Affiliations:** ^1^Department of Neurosurgery, The Second Hospital of Jilin University, Changchun, China; ^2^Department of Neurosurgery (Neurotrauma), The First Hospital of Jilin University, Changchun, China

**Keywords:** ethylene oxide, neurofilament light chain, HbEO, epidemiology, NHANES

## Abstract

**Background:**

Ethylene oxide (EO) is a widely used industrial chemical recognized for its health risks, potentially posing threats to human health, including neurotoxicity, cardiovascular damage, and carcinogenic effects. Neurofilament light chain (NfL) is a protein released into the blood following axonal damage. To date, no studies have investigated the relationship between EO exposure and NfL levels. Therefore, we selected 5,902,013–2014 National Health and Nutrition Examination Survey (NHANES) participants to examine the relationship between blood EO levels and serum NfL concentrations.

**Methods:**

According to the data from NHANES, this cross-sectional study used multiple regression analysis, subgroup analysis, and smooth curve fitting to explore the relationship between Ethylene oxide and Neurofilament light chain.

**Results:**

The results of the present study indicate a positive association between EO exposure and NfL concentrations. Greater EO exposure was correlated with increased serum NfL concentrations in the fully adjusted model [*β* = 0.28, 95% CI (0.16, 0.40)]. Each additional unit of EO exposure was linked to a 0.28-unit increase in the serum NfL level. Additionally, IN sensitivity analysis by changing EO exposure from a continuous variable to a categorical variable. The serum NfL concentrations increased with increasing tertiles of EO levels. Compared with the lowest tertile, the highest EO exposure tertile was associated with a 0.28-unit increase in serum NfL concentrations (*β* = 0.28, 95% CI 0.16, 0.40; P for trend = 0.0138).

**Conclusion:**

Our results indicate a link between increased EO levels and higher serum NfL concentrations in a sample of US adults aged 20 years and older. Although the directionality and clinical significance of this observation remain uncertain, our results emphasize the importance of conducting additional studies to investigate the possible causes and neurological effects of exposure to EO in adults.

## Introduction

Ethylene oxide (EO) is manufactured worldwide and mainly serves as a sterilizing agent for medical equipment and as a key component in the manufacturing of various chemicals (1). It is also utilized for the fumigation of foods (such as spices and nuts) and cosmetics (2). Occupational exposure to EO can occur in workplaces where EO is produced or used (3). Furthermore, EO can be produced when petroleum, natural gas, coal, and tobacco items are burned. EO is in a gaseous state at room temperature, which means that inhalation is the main way in which the body is exposed to this substance. The general population is exposed primarily to EO primarily through contaminated air, cigarette smoke, and automobile exhaust (4). Past research has indicated that individuals who smoke experience notably greater exposure to EO than those who do not smoke (5). Previous studies have indicated a correlation between occupational exposure to EO and the levels of hemoglobin adducts (HbEOs) in workers (6). EO is a commonly utilized alkylating substance that interacts with valine in hemoglobin to produce HbEO (N-(2-hydroxyethyl)valine). Hemoglobin adducts(HbEOs) can be detected with high efficiency and sensitivity and are thus widely used to evaluate EO exposure (7). Several datasets have demonstrated a correlation between occupational EO exposure and HbEO levels (8). Excessive exposure to EO, a highly reactive and volatile organic compound, can increase the likelihood of developing cardiovascular diseases, cancer, and neurological damage (9–11). In laboratory settings, EO have been shown to inhibit creatine kinase and induce axonal damage, potentially leading to a range of neurological disorders (12). Furthermore, prior research has indicated varying degrees of cognitive impairment in individuals exposed to EO (13, 14). Additionally, case reports have documented the manifestation of Parkinsonian symptoms following exposure to EO (15). The extensive utilization of EO has raised substantial concerns about their possible negative impacts on human health and the ecosystem. The widespread use of EO has raised significant concerns about its potential adverse effects on human health and the environment. Consequently, this topic remains a subject of considerable controversy and ongoing research (16).

As a neuron-specific marker of nerve injury, elevated levels of NfL can be seen in a variety of conditions involving nerve axon injury in the central and peripheral nervous systems and is considered a promising biomarker for neurodegenerative diseases (17). Higher levels of serum NfL are considered a marker for central-peripheral distal axonopathy disease and are correlated with more severe neurological damage and worse clinical outcomes in patients with conditions such as multiple sclerosis, Parkinson’s disease, and Alzheimer’s disease (18). Previous studies have indicated that NfL concentrations are associated with aging and cognitive decline (19). Additionally, serum NfL levels may reflect the prognosis of patients with traumatic brain injury (TBI), aiding in the prediction of long-term outcomes (20, 21). The diagnostic potential of NfL in clinical practice should be considered alongside other neurological conditions. NfL concentrations in cerebrospinal fluid and blood are altered in central nervous system disorders and are associated with specific disease features. In addition, as a quantitative marker of active axonal damage, increased NfL levels may offer valuable prognostic information across a range of neurological diseases.

To enhance our understanding of the connection between EO levels and biomarkers indicating neuronal damage in adults, we retrieved data from individuals who participated in the 2013–2014 National Health and Nutrition Examination Survey (NHANES), which offered accessible information on EO exposure and serum NfL concentrations. The aim of this study was to assess the potential correlation between EO exposure and serum NfL levels in a representative US population.

## Materials and methods

### Study population

The NHANES, conducted by the National Center for Health Statistics (NCHS), is a survey that represents the entire nation and is conducted in a single period (22). The survey utilizes a comprehensive strategy that involves physical exams and interviews to gather information on demographics, socioeconomic status, and health (23). The survey occurs every 2 years, and except confidential data, all pertinent information can be found on the National Center for Health Statistics website. The recruitment process followed the ethical guidelines outlined in the survey protocol, with the NHANES protocol obtaining ethical approval from the Ethics Review Board of the NCHS at the CDC. Written informed consent was obtained from all participants.

**Figure 1:**
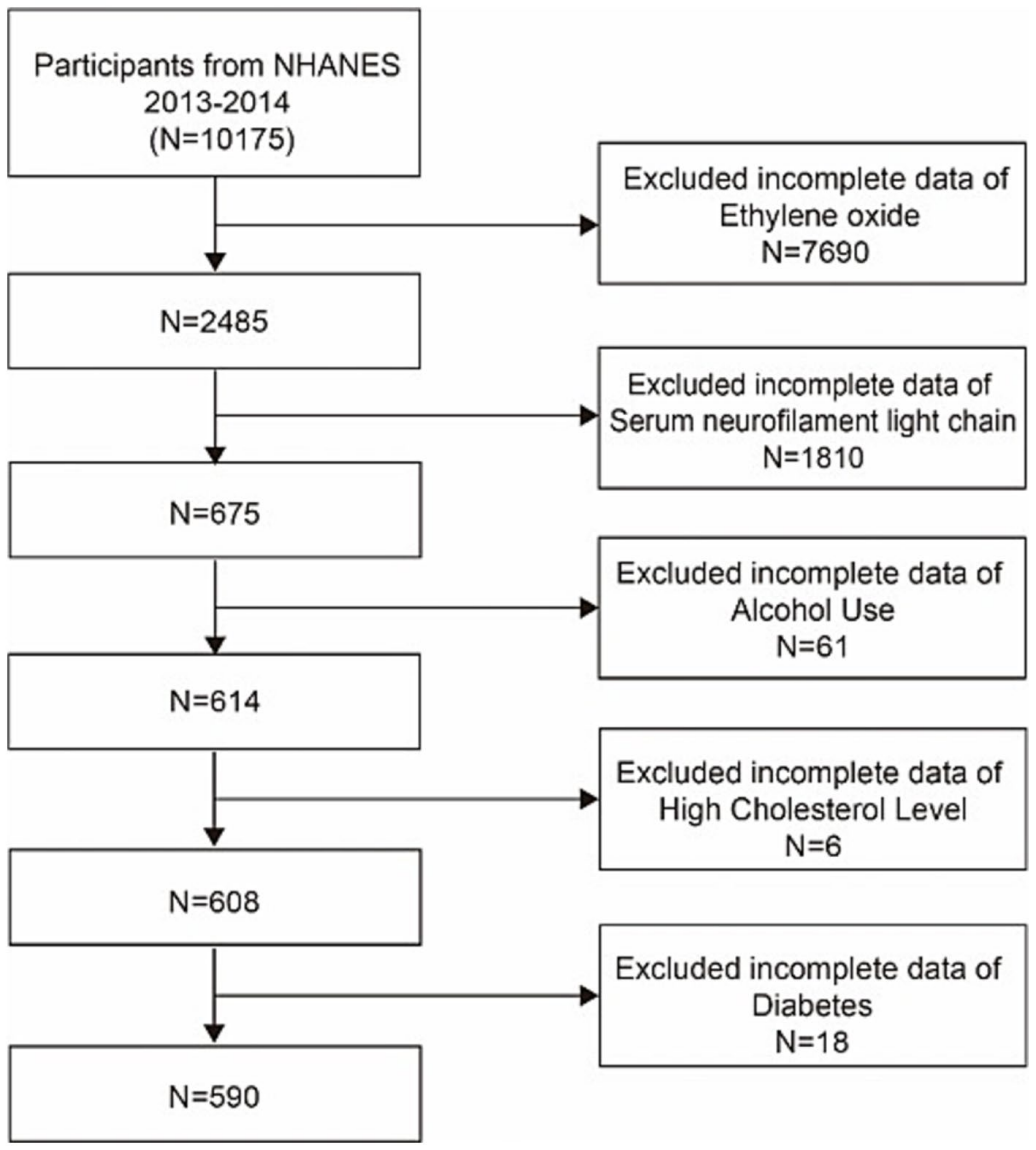
Flowchart of participant selection. NHANES, National Health and Nutrition Examination Survey.

Data for this research were collected from a representative sample via a cluster, stratified, multistage sampling technique, as well as a cross-sectional study approach. We utilized data from the 2013–2014 NHANES cycle, which measured the EO concentration in subjects and assessed serum NfL concentrations in a subset of participants aged 20–75 years. Among the 10, 175 participants initially involved in this survey, those with missing HbEO levels (*n* = 7,690), a lack of serum NfL data (*n* = 1,810), and those missing data on hyperlipidemia (*n* = 6) and diabetes (*n* = 18) status were excluded. We also assessed various social habits, excluding some participants with missing data on alcohol consumption (*n* = 61) and BMI (*n* = 4). Ultimately, our study included 590 participants, as illustrated in Figure 1.

### Measurement of EO in hemoglobin

After appropriate processing of red blood cell samples, they were transported under low-temperature conditions (approximately −30°C) to the NCEH for comprehensive analysis. Hemoglobin adducts were evaluated by measuring overall hemoglobin levels. To achieve this, the reaction mixture was carefully combined with the samples, followed by separation of the Edman degradation products. MS was employed to measure HbEO levels in whole blood or red blood cells by analyzing N-[2-carbamoyl ethyl] valine and N-[2-hydroxy carbamoyl ethyl] valine EO adducts. The biological half-life of HbEO is approximately 4 months, which makes it a useful substitute indicator for evaluating exposure to EO (24). The findings were measured and reported as picomoles of adducts per gram of hemoglobin. Commercially available hemoglobin assay kits were used to measure the hemoglobin levels used for the modification of the Edman reaction. HbEO concentrations in the bloodstream were measured in pmol/g of hemoglobin, with a minimum detectable level of 12.90 pmol/g of hemoglobin. Detailed information on the modified Edman method for detecting HbEO can be found in the NHANES Laboratory/Medical Technologist Procedures Manual. Detailed measurements are accessible via the following website.^1^ All tests met the accuracy and precision performance standards set by the NCEH Laboratory Science Division for quality control and assurance.

### Serum NfL measurement

Serum NfL levels were measured via a high-sensitivity immunoassay developed by Siemens Healthineers. All procedures were conducted on the fully automated Atellica Immunoassay System. The levels of serum NfL were measured using a high-sensitivity immunoassay developed by Siemens Healthineers. Every process was carried out using the completely automated Atellica immunoassay platform. The detection rate for serum NfL was 98.4%. Values below the quantification limit were approximated via an estimated value (the quantification limit divided by the square root of 2). The analytical technique can be accessed at the NHANES site and is thoroughly explained in our additional section (CDC 2022).

### Covariates

In this study, we incorporated age (continuous), gender (categorical), race/ethnicity (categorical), the poverty–income ratio (PIR; categorical) was the ratio of household income to the established poverty line and is used to assess socio-economic status and household income levels, alcohol consumption status (categorical), body mass index (BMI; continuous), and hypertension (categorical), diabetes (categorical), and hyperlipidemia (categorical) status as factors to consider. For more in-depth details about the gathered covariates, refer to the NHANES database.

### Statistical analysis

For descriptive analysis, data description and statistical analysis were performed via a composite weighting method. Categorical data are represented as weighted percentages, whereas continuous data are presented as the means with standard deviations. Natural logarithm (ln) transformations were applied to the EO and serum NfL levels to approach a normal distribution. In complex sample general linear models, serum NfL was the dependent variable and EO was the independent variable. Variations between participants categorized by HbEO level tertiles were evaluated through weighted standard t tests for continuous factors and weighted chi-square tests for categorical factors.

Three regression models were developed: Model 1 was not adjusted for confounding factors; Model 2 was adjusted for included age, gender and race, and Model 3 was additionally adjusted for education level, the family income–poverty ratio, and hypertension, diabetes, hyperlipidemia, and alcohol consumption status. Additionally, smooth curve fitting was used to examine whether the relationship between EO exposure and serum NfL concentrations was linear. If nonlinear, threshold effect analysis was used to provide intervals and calculate thresholds. Missing values for categorical variables on the basis of existing data were represented by other means, whereas missing values for continuous variables were interpolated by means. Stratification factors such as gender, age, BMI, and hypertension and diabetes status were considered in the subgroup analysis to examine the relationships between EO exposure and serum NfL concentrations. EmpowerStats^2^ was used for all the analyses. A two-tailed *p*-value less than 0.05 was considered to indicate statistical significance.

## Results

### Baseline characteristics

The results of this cross-sectional analysis involving 590 NHANES subjects with an averageage of 46.95 ± 15.61 years are displayed in Figure 1. The study sample sex proportion was 49.84% men and 50. 16% women. The baseline characteristics of the HbEO levels in our study are presented in Table 1. Gender, household income, race, education level, hypertension, and BMI significantly varied (*p* < 0.05) among the three distinct HbEO levels. Individuals in the group with greater exposure to EO were at greater risk of having hypertension, high BMI, and diabetes than those in the group with the lowest EO exposure (all *p* < 0.05). There were no notable distinctions observed among the groups regarding age or hyperlipidemia status (*p* < 0.05; Table 1). Serum neurofilament light chain protein had a mean of 2.48, a standard deviation of 0.68, and a beta of 0.28 occupied about one-tenth of the mean. Serum NfL levels were elevated in patients compared to the healthy population, and the greater the degree of cognitive impairment, the higher the serum NfL levels.

**Table 1:** Characteristics of study population.

Characteristics	Total (*n* = 590)	Q1 (*n* = 196)	Q2 (*n* = 197)	Q3 (*n* = 197)	Value of *p*
Age (years)	45.92 ± 15.50	46.22 (15.00)	46.66 (15.32)	44.75 (16.21)	0.4682
Gender (%)					0.1248
Male	51.05	45.67	54.39	54.25	
Female	48.95	54.33	45.61	45.75	
Race/ethnicity (%)					<0.0015*
Mexican American	10.32	11.04	14.57	4.94	
Other Hispanic	6.95	6.28	9.32	5.30	
Non-Hispanic white	68.37	72.07	61.81	70.67	
Non-Hispanic black	8.41	4.79	7.02	14.40	
Other race	5.95	5.82	7.29	4.70	
PIR (%)					0.0006^*^
<1.3	22.74	19.22	18. 11	32.03	
≥1.3, <3.5	34.36	33.21	32.91	37.31	
≥3.5	42.90	47.57	48.98	30.66	
Education level (%)					<0.0001^*^
Less than 9th grade	4.55	3.25	5.07	5.64	
9-11th grade	9.50	5.04	8.96	15.65	
High school graduate	19.93	16.32	16.02	28.57	
Some college or AA degree	34.79	37.32	33.46	33.02	
College graduate or above	31.22	38.08	36.49	17. 12	
High blood pressure (%)					0.0526
Yes	31.41	32.19	25.26	36.92	
No	68.59	67.81	74.74	63.08	
High cholesterol level (%)					0.1229
Yes	33. 14	33.82	37.59	27.62	
NO	66.86	66.18	62.41	72.38	
Diabetes (%)					0.1718
Yes	9.94	7.94	13.27	8.93	
No	90.06	92.06	86.73	91.07	
Alcohol use (%)					0.0112^*^
Yes	80.69	78.65	76.26	87.93	
No	19.31	21.35	23.74	12.07	
BMI (kg/m^2^; %)					0.0230^*^
<25.0	32.96	26.58	34.52	39.28	
≥25.0	67.04	73.42	65.48	60.72	

The *p*-value was calculated by weighted linear regression model. % for categorical variables: the *p*-value was calculated by a weighted chi-square test. BMI, body mass index; PIR, poverty income ratio. *Symbol to indicate some groups that are statistically significant and contribute to the argument of the article’s results for ease of reading.

### Associations between EO exposure and serum NfL protein concentrations

The results of the present study indicate a positive association between EO exposure and NfL concentrations (Table 2). Greater EO exposure was correlated with increased serum NfL concentrations in the fully adjusted model [*β* = 0.28, 95% CI (0.16 ~ 0.40)]. Each additional unit of EO exposure was linked to a 0.28-unit increase in the serum NfL level. Additionally, we conducted a sensitivity analysis by changing EO exposure from a continuous variable to a categorical variable (tertile). The serum NfL concentrations increased with increasing tertiles of EO levels: model 1 (OR = 0.29, 95% CI = 0.15 ~ 0.42, *p* < 0.0001), model 2 (OR = 0.31, 95% CI = 0.19 ~ 0.42, *p* < 0.0001), model 3 (OR = 0.28, 95% CI = 0.16 ~ 0.40, *p* < 0.0001). Compared with the lowest tertile, the highest EO exposure tertile was associated with a 0.28-unit increase in serum NfL concentrations (*β* = 0.28, 95% CI 0.16, 0.40; *p* for trend = 0.0138; Table 2).

**Table 2:** The association between blood ethylene oxide levels and serum neurofilament light chain.

Variable	Crude model		Model I		Model II	
	OR (95%CI)	*p*-value	OR (95%CI)	*p*-value	OR (95%CI)	*p*-value
EO exposure						
Q1	Ref		Ref		Ref	
Q2	0. 14 (0.01, 0.27)	0.0412	0. 13 (0.02, 0.24)	0.0236	0. 14 (0.03, 0.26)	0.0121
Q3	0.29 (0. 15, 0.42)	<0.0001^*^	0.31 (0. 19, 0.42)	<0.0001^*^	0.28 (0. 16, 0.40)	<0.0001^*^

Coarse model, no covariate was adjusted. Model 1, confounding factors was not adjusted. Model 2, Sex, age, race were adjusted. Model 3, Sex, age, race, education, PIR, insurance, drinking, BMI, diabetes, hypertension, hyperlipidemia were adjusted. *Symbol to indicate some groups that are statistically significant and contribute to the argument of the article’s results for ease of reading.

### Subgroup analysis

**Figure 2:**
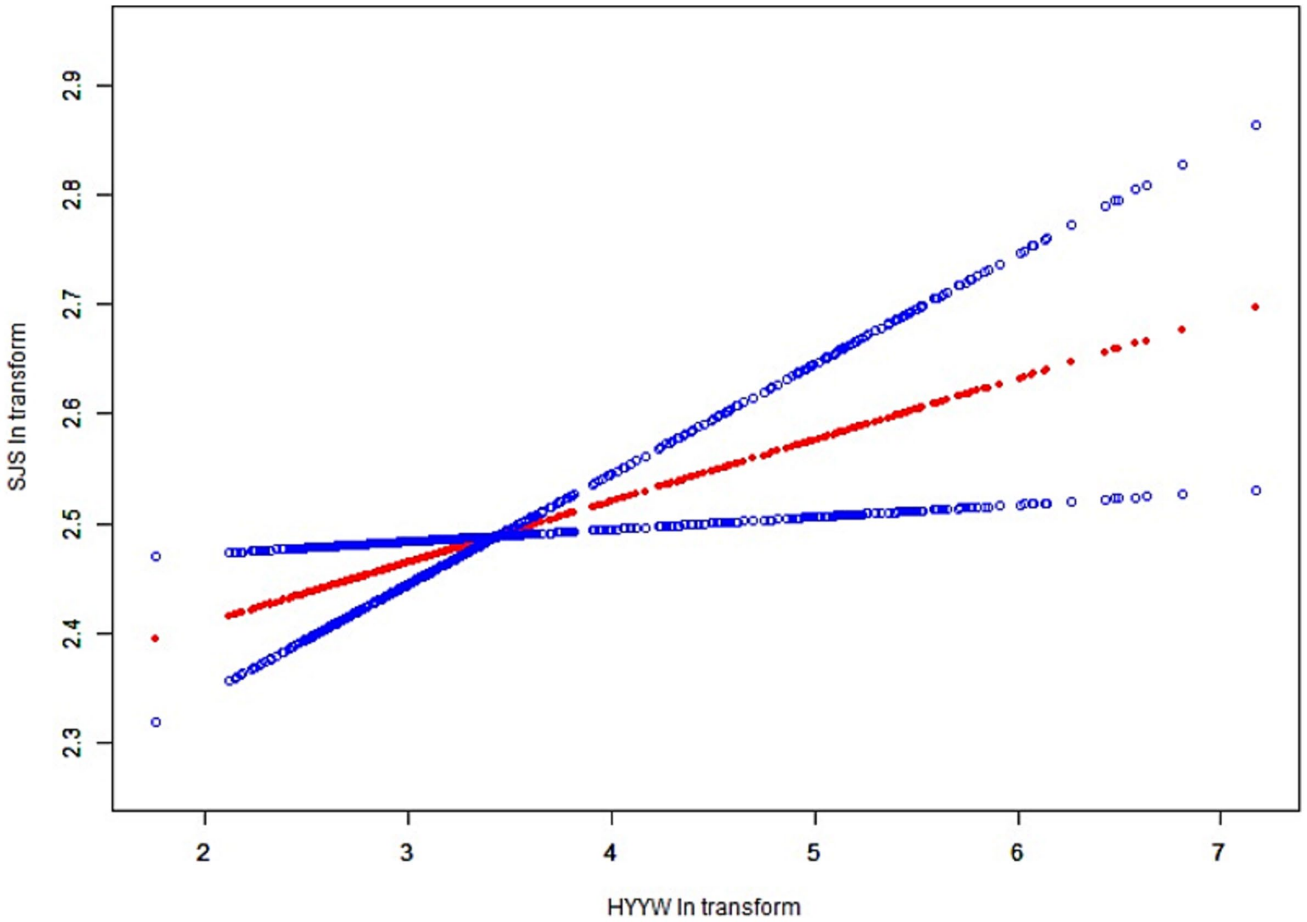
The nonlinear relationship was depicted as a smooth curve fit. The red line represents the curve fit of EO to Nfl and the blue line represents the execution interval of the curve fit.

In all subgroup analyses, the effects of HbEO on the serum NfL concentration were consistent across the race, gender, BMI, and alcohol consumption, hypertension, hyperlipidemia and diabetes status subgroups, with no significant interactions observed. However, a positive correlations were observed in the education level and PIR subgroups (*p* < 0.05). The results of the subgroup analysis of the relationships between HbEO levels and serum NfL level are presented in Table 3. In addition, The nonlinear relationship was depicted as a smooth curve fit (Figure 2).

**Table 3:** Subgroup analysis of the association between blood ethylene oxide levels and serum neurofilament light chain.

Subgroup	HbEO (pmol/g Hb)		Q3 *OR* (95% *CI*)	P for interaction
	Q1 *OR* (95% *CI*)	Q2 *OR* (95% *CI*)		
Gender				0.7921
Male	1.00	0. 18 (0.02, 0.34)	0.26 (0.09, 0.43)	
Female	−1.25 (−2.03, −0.46)	−1. 10 (−1.87, −0.32)	−0.93 (−1.70, −0. 16)	
Race/ethnicity				0.1659
Mexican American	1.00	0.08 (−0.26, 0.43)	−0. 17 (−0.70, 0.35)	
Other Hispanic	−0.01 (−2. 14, 2. 12)	0.34 (−1.83, 2.51)	0.41 (−1.77, 2.60)	
Non-Hispanic white	0.26 (−1.31, 1.84)	0.43 (−1. 14, 2.01)	0.62 (−0.95, 2. 19)	
Non-Hispanic black	−0.82 (−2.72, 1.07)	−1. 15 (−3.04, 0.74)	−1.03 (−2.87, 0.82)	
Other race	0. 17 (−2.29, 2.64)	0.36 (−2.02, 2.74)	0.21 (−2.20, 2.62)	
Education level				0.0389^*^
Less than 9th grade	1.00	−0.06 (−0.68, 0.55)	0.03 (−0.65, 0.71)	
9-11th grade	1. 10 (−1.20, 3.41)	1. 16 (−1. 16, 3.48)	1.04 (−1. 19, 3.27)	
High school graduate	0.24 (−1.76, 2.24)	0.88 (−1.09, 2.85)	0.63 (−1.36, 2.63)	
Some college or AA degree	0.08 (−1.85, 2.01)	0. 17 (−1.76, 2. 10)	0.28 (−1.65, 2.21)	
College graduate or above	1.22 (−0.77, 3.21)	1.26 (−0.73, 3.25)	1.48 (−0.50, 3.46)	
High blood pressure				0.5993
Yes	1.00	0. 19 (−0.03, 0.42)	0.37 (0. 16, 0.57)	
No	−0.06 (−0.96, 0.84)	0.08 (−0.82, 0.98)	0. 18 (−0.72, 1.08)	
High cholesterol level				0.8619
Yes	1.00	0. 13 (−0.07, 0.32)	0.21 (−0.01, 0.44)	
NO	0. 11 (−0.78, 1.00)	0.27 (−0.62, 1. 16)	0.40 (−0.49, 1.28)	
Diabetes				0.4778
Yes	1.00	−0.05 (−0.47, 0.37)	−0.02 (−0.55, 0.51)	
No	−0.08 (−1.83, 1.67)	0.08 (−1.67, 1.83)	0.21 (−1.54, 1.96)	
Alcohol use				0.4448
Yes	1.00	0. 16 (0.03, 0.29)	0.31 (0. 18, 0.44)	
No	−0. 12 (−1.06, 0.81)	0.01 (−0.93, 0.96)	−0.03 (−0.95, 0.89)	
BMI (kg/m2)				0.1282
<25.0	1.00	0.27 (0.06, 0.47)	0.44 (0.21, 0.67)	
≥25.0	0.73 (−0.26, 1.72)	0.83 (−0. 16, 1.82)	0.91 (−0.08, 1.90)	
PIR				0.0158^*^
<1.3	1.00	0.24 (−0.03, 0.50)	0.21 (−0.04, 0.46)	
≥1.3, <3.5	0.43 (−0.62, 1.48)	0.59 (−0.48, 1.65)	0.96 (−0.08, 2.00)	
≥3.5	0.55 (−0.37, 1.46)	0.72 (−0. 18, 1.62)	0.67 (−0.26, 1.60)	

95% CI, 95% confidence interval; OR, odds ratio; PIR, poverty income ratio; BMI, body mass index. The strata variable was not included when stratifying by itself. *Symbol to indicate some groups that are statistically significant and contribute to the argument of the article’s results for ease of reading.

## Discussion

This cross-sectional study included 590 participants. We observed a positive correlation between EO exposure and serum NfL concentrations. This association was not significantly impacted by sex, age, BMI, or hypertension, diabetes, or hyperlipidemic status. Our findings suggest that elevated EO levels are associated with increased serum NfL concentrations in the general adult population, and this association remains significant across most subgroups. This study is the first to suggest a connection between exposure to ethylene oxide and indicators of nerve damage in the overall adult population of the United States. However, whether the elevation in serum NfL levels due to EO exposure leads to clinically significant neurological diseases remains uncertain. This study is noteworthy because it represents US adults aged 20 years and older and utilizes reliable and comprehensive data from the NHANES database. The results underscore the possible harmful effects of EO on the nervous system, underscoring the need to monitor and reduce its negative health consequences. To our knowledge, this study is the first to explore the connection between EO exposure and serum NfL levels.

EO is often cited as a major hazard for staff at sterilization facilities, especially those involved in the sterilization of medical devices. It is important to consider the potential danger of exposure to EO for the general public, which may come from sources such as vehicle emissions, smoking, and proximity to facilities that utilize EO, resulting in exposure to volatile organic compounds (5). Research has shown that exposure to EO can cause genetic mutations and damage to genes, leading to a range of negative health consequences (25, 26). A study cohort utilizing data from the Toxic Substances Release Inventory of the US Environmental Protection Agency reported a greater likelihood of developing *in situ* breast cancer in individuals exposed to environmental EO, with no elevated risk for invasive breast cancer or non-Hodgkin lymphoma (10). Occupational EO exposure may increase the risk of mortality from lymphohematopoietic malignancies (27). For nonmalignant diseases, elevated HbEO levels are associated with lower high-density lipoprotein cholesterol concentrations and increased diabetes risk (5). Populations with elevated HbEO levels presented also presented increased rates of hypertension and elevated diastolic blood pressure (28). Previous case reports, including those involving healthcare workers, have documented peripheral and central nervous system dysfunction following EO exposure, suggesting that other healthcare workers may face similar risks (15, 29). Reported abnormal nerve conduction velocities in several workers after EO gas exposure, with no change in abnormal nerve conduction studies in patients who continued to work at lower exposure levels, whereas those who were removed from exposure showed improvement in conduction abnormalities (30). The characteristic pathology caused by EO is myelinated fibers distal axonal degeneration, with the mean cross-sectional area of axons reduced by approximately 53% compared to controls. These changes include axonal swelling and vesicle accumulation, which suggest impaired axonal transport. Since these toxins react preferentially with protein sulfhy dry functions, it is hypothesized that critical sulfhydryl groups on transport-related proteins lead to reduced nutrient delivery to distal axons and result in degeneration, which in turn leads to Nfl release following neurofilament disruption. Neurofilaments, which are abundant in developed myelinated axons, are made up of light, medium, and heavy chains (31). Following damage to neurons from different risk factors, NfL is discharged into the cerebrospinal fluid (CSF) and later enters the circulation. The levels of NfL in cerebrospinal fluid and blood have been demonstrated to be valuable and easily accessible indicators for tracking the advancement and severity of a disease (32). Neurosurgeons often pay close attention to serum NfL levels because they reflect the activity of certain neurological diseases, including Alzheimer’s disease, stroke, small vessel disease, and head trauma (19, 33, 34) used intraepidermal nerve fiber density quantification to evaluate the occurrence and progression of peripheral neurotoxicity and reported that serum NfL levels are correlated with the severity of structural and functional changes in axons (35). Therefore, NfL may serve as a useful biomarker for axonal injury, tracking the occurrence and severity of axonal degeneration. Studies conducted on mice have demonstrated distal axonal damage in rats following exposure to EO (36). Reported that central–peripheral distal axonopathy of primary sensory neurons in the gastrocnemius nerve and fine fascicles was induced in experimental rats exposed to 250 ppm EO (37). Additionally, reported a notable link between exposure to EO and increased incidences of mononuclear cell leukemia, peritoneal mesothelioma, and mixed-cell brain gliomas in rats (38). Another experiment indicated that EO could inhibit creatine kinase (CK) in the brain and spinal cord, causing central-peripheral distal axonopathy (39). CK is also a component transported by axons and is related to ATP synthesis. Therefore, suppressing CK function could contribute to the development of encephalopathy and distal axonopathy caused by neurotoxins. A potential mechanism underlying this effect is that ethylene oxide (EO) may inhibit creatine kinase (CK) activity in the rat brain and spinal cord, thereby disrupting synaptic ATP production. ATP is essential for the enzymatic phosphotransfer network and plays a critical role in maintaining cellular energy homeostasis. Beyond its function as a synaptic transmitter in the central nervous system (40), ATP is also crucial for providing energy to neurons, ensuring proper nervous system function (41). Consequently, exposure to ethylene oxide impairs ATP synthesis, potentially resulting in neurological symptoms. These findings indicate that prolonged exposure to EO could result in significant brain damage. Our research reveals a new association between EO exposure and Nfl concentrations, indicating that EO exposure could lead to damage to neuronal axons and the release of NfL into the blood of the overall adult population in the United States.

Our study has several strengths. First, we used data that represent the entire nation to fully elucidate the effects of exposure to environmental odors and how EO exposure correlates with the levels of serum NfL. This groundbreaking research delves into the correlation between exposure to EO and serum NfL levels, shedding light on the possible neurological effects of this common industrial chemical. However, some limitations warrant consideration. The limited number of participants with NfL data from NHANES 2013–2014 hinders the ability to perform thorough analyses and strengthen the credibility of the results. Although we have adjusted for a number of potential covariates, many factors still influenced this study, and we cannot completely rule out the influence of other potential confounders (e.g., history of occupational exposure and genetic susceptibility). In addition, the cross-sectional study design prevents us from making inferences about the causal relationship between EO exposure and Nfl, and its only possible to explore the relationship between EO exposure and Nfl to provide clues for the study. Future studies that use longitudinal research methods and incorporate a wider variety of neurobiological indicators will be beneficial for investigating these intricate connections. Third, our study population consisted of US adults, so we cannot assume that these findings apply to other age groups and countries. Given that EO exposure can fluctuate over time, depending solely on one measurement may not completely reflect the cumulative exposure and its influence on serum NfL levels.

## Conclusion

To summarize, our results indicate a link between increased EO levels and higher serum NfL concentrations in a sample of US adults aged 20 years and older. Although the directionality and clinical significance of this observation remain uncertain, our results emphasize the importance of conducting additional studies to investigate the possible causes and neurological effects of exposure to EO in adults. The results of this study may have significant consequences for public health policies and regulatory choices concerning the utilization of EO.

## Data Availability

Publicly available datasets were analyzed in this study. This data can be found here: https://wwwn.cdc.gov/nchs/nhanes/Default.aspx.

## Footnotes

^1^ https://wwwn.cdc.gov/Nchs/Nhanes/2013-2014ETHOX_H.htm

^2^ http://www.empowerstats.com
